# Invasion biology in non-free-living species: interactions between abiotic (climatic) and biotic (host availability) factors in geographical space in crayfish commensals (Ostracoda, Entocytheridae)

**DOI:** 10.1002/ece3.897

**Published:** 2013-12-03

**Authors:** Alexandre Mestre, Josep A Aguilar-Alberola, David Baldry, Husamettin Balkis, Adam Ellis, Jose A Gil-Delgado, Karsten Grabow, Göran Klobučar, Antonín Kouba, Ivana Maguire, Andreas Martens, Ayşegül Mülayim, Juan Rueda, Burkhard Scharf, Menno Soes, Juan S Monrós, Francesc Mesquita-Joanes

**Affiliations:** 1 Department of Microbiology and Ecology, Institut Cavanilles de Biodiversitat i Biologia Evolutiva, University of ValenciaBurjassot, E-46100, Spain; 2 Cessy Angling AssociationCessy, F-01170, France; 3 Department of Biology, Istanbul UniversityVezneciler, 34134, Turkey; 4 Ahern Ecology Ltd.Wilton, SP2 0HE, U.K; 5 Institut für Biologie, Pädagogische Hochschule KarlsruheKarlsruhe, 76133, Germany; 6 Department of Zoology, University of ZagrebZagreb, HR-10000, Croatia; 7 Faculty of Fisheries and Protection of Waters, University of South BohemiaVodňany, 389 25, Czech Republic; 8 Ellhornstrasse 21, Bremen, D-28195, Germany; 9 Naturalis Biodiversity CenterLeiden, 2333 CR & Bureau Waardenburg, Culemborg, 4100 AJ, The Netherlands

**Keywords:** Biological invasions, BAM diagrams, ecological niche models, host availability

## Abstract

In invasion processes, both abiotic and biotic factors are considered essential, but the latter are usually disregarded when modeling the potential spread of exotic species. In the framework of set theory, interactions between biotic (*B*), abiotic (*A*), and movement-related (*M*) factors in the geographical space can be hypothesized with BAM diagrams and tested using ecological niche models (ENMs) to estimate *A* and *B* areas. The main aim of our survey was to evaluate the interactions between abiotic (climatic) and biotic (host availability) factors in geographical space for exotic symbionts (i.e., non-free-living species), using ENM techniques combined with a BAM framework and using exotic Entocytheridae (Ostracoda) found in Europe as model organisms. We carried out an extensive survey to evaluate the distribution of entocytherids hosted by crayfish in Europe by checking 94 European localities and 12 crayfish species. Both exotic entocytherid species found, *Ankylocythere sinuosa* and *Uncinocythere occidentalis*, were widely distributed in W Europe living on the exotic crayfish species *Procambarus clarkii* and *Pacifastacus leniusculus,* respectively. No entocytherids were observed in the remaining crayfish species. The suitable area for *A. sinuosa* was mainly restricted by its own limitations to minimum temperatures in W and N Europe and precipitation seasonality in circum-Mediterranean areas. *Uncinocythere occidentalis* was mostly restricted by host availability in circum-Mediterranean regions due to limitations of *P. leniusculus* to higher precipitation seasonality and maximum temperatures. The combination of ENMs with set theory allows studying the invasive biology of symbionts and provides clues about biogeographic barriers due to abiotic or biotic factors limiting the expansion of the symbiont in different regions of the invasive range. The relative importance of abiotic and biotic factors on geographical space can then be assessed and applied in conservation plans. This approach can also be implemented in other systems where the target species is closely interacting with other taxa.

## Introduction

### Biotic and abiotic factors in invasion processes

Dramatic impacts of alien species on invaded ecosystems have prompted interest to scientifically understand invasion processes in order to prevent their harmful effects (Strayer et al. 2006; Young and Larson 2011). Invasive species have a combination of attributes that facilitate their arrival and establishment in a novel region (Sol 2007; Karatayev et al. 2009). But several external factors are also involved in an invasion success, usually classified into abiotic, biotic, and dispersal factors. Although some authors give more importance to dispersal factors such as propagule pressure in accounting for the success or failure of an invasion event (e.g., Lockwood et al. 2005), abiotic and biotic factors have been shown as important elements in invasion biology.

The role of the abiotic conditions in invasion biology is evident, and physical suitability for an invader obtained from environmental predictors, mainly climatic, has been considered as good predictor of invasibility (Williamson 1996). Several studies also show that spatial and temporal heterogeneity and physical disturbances, usually related to abiotic conditions (like climatic or geographical), may facilitate the establishment of invasive species (Melbourne et al. 2007). Another example of the importance of abiotic factors in invasion biology is the effect of climate change on the invasion processes (Hellmann et al. 2008; Rahel and Olden 2008).

In spite of the wide use of climatic conditions to predict the regions susceptible to be invaded by exotic species, biotic interactions have also been shown as important elements limiting the species distributions (Guisan and Thuiller 2005). Indeed, biotic interactions are considered a key factor in biological invasions (White et al. 2006; Roy and Handley 2012). Biotic factors such as community complexity, the existence or absence of enemies (predators, competitors, parasites, and pathogens), and mutualisms or commensalisms with other species may facilitate or hamper the establishment of an invader in a novel area (Mooney and Cleland 2001; Sakai et al. 2001; Prenter et al. 2004; Davis 2009; Engelkes and Mills 2011). For example, the Enemy Release Hypothesis proposes a facilitation of the invasion success due to loss of negative interactions from the native range, including competition, predation, or parasitism, during the early invasive stages of the displacement to the novel area (Sax and Brown 2000; Torchin et al. 2003; Roy et al. 2011). But those symbionts that get to remain with the exotic species during the invasive process have also an important role. Host jump, a key element in the evolution of non-free-living organisms (Poulin 2007), is also essential in invasion biology. An invasion event offers new biogeographic and evolutionary opportunities to the symbionts accompanying an invasive host. The process of symbiont transmissions from invasive to native hosts, also called “spillover” (Kelly et al. 2009), is considered an important threat for native species conservation (Roy and Handley 2012; Strauss et al. 2012). [NB: This work employs the term “symbiosis” with its broad meaning of organisms living in association, including positive (mutualism), negative (parasitism), and neutral (commensalism) interactions, following Sapp (1994). The terms “symbiont” and “non-free-living species” are employed for a smaller organism living in symbiosis with a larger species, termed the “host”].

### The ecological niche in set theory and BAM diagrams

According to the niche concept proposed by Hutchinson (1957), “an n-dimensional hypervolume is defined, every point in which corresponds to a state of the environment which would permit the species S_l_ to exist indefinitely.” The potential niche is the range of environmental conditions available in the geographical space associated with positive intrinsic growth rates. The realized niche is the portion of the potential niche without biotic and/or dispersal constrictions. We want to highlight the distinction between the environmental space, linked to the niche concept, and the geographical space, composed of grid cells covering a particular region, associated with the geographical distribution of species (Peterson et al. 2011).

Based on the application of set theory (Hrbacek and Jech 1999) to niche concepts, BAM diagrams (Soberón and Peterson 2005) offer a framework to configure different hypothetical interactions between biotic (*B*), environmental or abiotic (*A*), and movement-related or dispersal (*M*) factors in the geographical space, which can be applied to invasion biology (Jiménez-Valverde et al. 2011). In this framework, *A* is the geographical area in which the environment is suitable at a given time, and where the intrinsic growth rate of the species would be positive; *B* is the geographical area where biotic interactions are favorable for species' existence, and *M* is the geographical area that is accessible to the species. In these models, the geographical area occupied by the species (*G*_*o*_) is that with suitable environmental conditions for species existence, favorable biotic interactions, and accessible for the species (A ∩ B ∩ M). Here, *A* represents the geographical area where the environmental conditions belong to the environmental space of the potential niche, and *G*_*o*_ is the projection of the realized niche in the geographical space. Therefore, the BAM diagrams link the environmental space of the niche theory with the geographical space of the species distributions.

**Figure 1:**
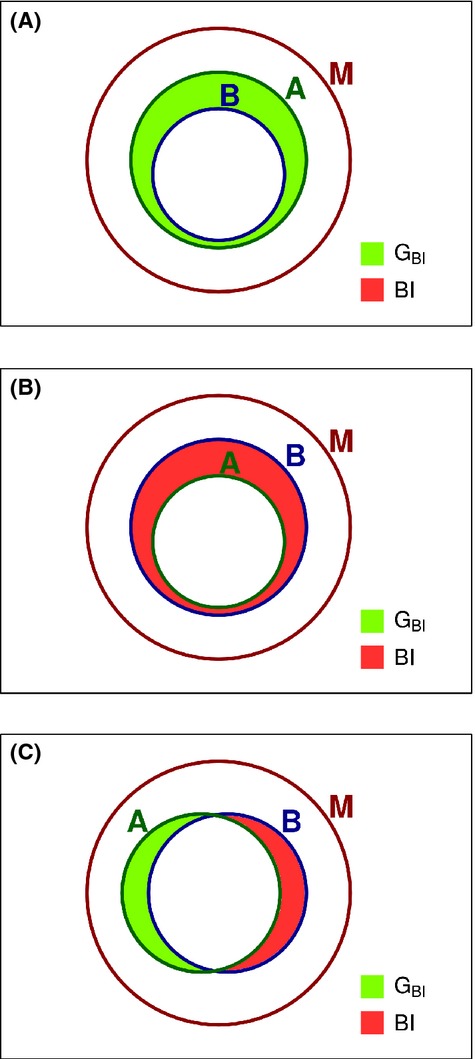
BAM diagrams adapted from Jiménez-Valverde et al. (2011) representing the three possible interactions between environmental and biotic factors in the geographical space of a species distribution model for invasive species when the species has no dispersal limitations ((*A* ∪ *B*) ⊂ *M*). Represented by circles, *A* is the geographical area with suitable environmental conditions, *B* the area where biotic interactions allow species existence, and *M* is the accessible area for the species. *G*_*BI*_ is the available geographical area with favorable environmental conditions, but inappropriate biotic conditions (*G*_*BI*_ = *A*\*B*) and *BI* the area with unsuitable environmental, but appropriate biotic conditions (*BI* = *B*\*A*) for the species. Within this model frame, the three possible interactions between *A* and *B* are as follows: (A) *A* includes *B* (*B* ⊂ *A* or (*A*\*B* ≠ ∅) ∧ (*B*\*A* = ∅)), (B) *B* includes *A* (*A* ⊂ *B* or (*A*\*B* = ∅) ∧ (*B*\*A* ≠ ∅)), and (C) a partial overlap between *A* and *B* ((*A*\*B* ≠ ∅) ∧ (*B*\*A* ≠ ∅)). Colors for *G*_*BI*_ and *BI* as in Fig. 5.

We can hypothesize the different possible interactions between *A* and *B* by means of BAM diagrams. Only three interactions are possible (Fig. 1): (1) *A* contains *B* (*B* ⊂ *A* or (*A*\*B* ≠ ∅) ∧ (*B*\*A* = ∅)), (2) *B* contains *A* (*A* ⊂ *B* or (*A*\*B* = ∅) ∧ (*B*\*A* ≠ ∅)), and (3) a partial overlap between *A* and *B* ((*A*\*B* ≠ ∅) ∧ (*B*\*A* ≠ ∅)). In a theoretical context in which there are no restrictions by accessibility (i.e., *M* contains *A* and *B*, (*A* ∪ *B*) ⊂ *M*), two areas of the BAM framework of Soberón and Peterson (2005) characterize the three cases: *G*_*BI*_ is the geographical area accessible and presenting favorable environmental conditions but inappropriate biotic conditions, and *BI* is the environmentally unsuitable but biotically appropriate area. In this theoretical context, *G*_*BI*_ is the portion of *A* that remains out of *B* (*G*_*BI*_ = *A*\*B*), and *BI* is the portion of *B* that does not coincide with *A* (*BI* = *B*\*A*); therefore, in the first case when *A* contains *B,* only *G*_*BI*_ (but not *BI*) will appear; in the second case when *B* contains *A,* only *BI* will appear; finally, in the third case of a partial overlap between *A* and *B*, both area types, *G*_*BI*_ and *BI*, will be present. So, *G*_*BI*_ and *BI* can be used to identify which model of interaction between *A* and *B* fits or is closer to the case of the exotic species analyzed, through the evaluation of their relative proportion. Moreover, they also represent areas where the species is specifically absent due to abiotic (*BI*) or biotic (*G*_*BI*_) factors, so that these factors are acting as specific barriers against the species expansion into those areas.

### Ecological niche models and set theory

Ecological niche models (ENMs) have proven useful in providing statistical tools to predict the environmentally suitable areas for the invasion by an exotic species (Thuiller et al. 2005), a practical approach that has been widely used recently (e.g., Reshetnikov and Ficetola 2011). The predictions are based on modeling the relation between species occurrence data and environmental predictors. Although biotic factors may also affect species distributions, most ENMs are based only on physical predictors because the high complexity of biotic interactions makes their inclusion in an ENM approach difficult. Nonetheless, some studies consider biotic interactions in their analyses, by adding biotic predictors or constraining the model predictions to the presence of interacting species (e.g., Heikkinen et al. 2007; Meier et al. 2010; Schweiger et al. 2012). Recently, novel techniques have incorporated biotic interactions into ENMs through modeling multispecies interactions by means of interaction matrices (Kissling et al. 2012). On the other hand, the application of ENMs to invasion biology is subject to methodological uncertainties derived from doing predictions across space and time. In this sense, the development of ensemble ENM techniques has represented a useful progress in order to assess the modeling uncertainty (Capinha and Anastácio 2011; Capinha et al. 2011).

ENMs can be applied in a theoretical framework of BAM models to analyze the interactions between *A* and *B* in the geographical space of exotic species that are strongly affected by a particular interaction with other species, for example, the dependence on the presence of a specific host, prey, or mutualist, or the absence of a particular predator or parasite. To do so, as we are not focused on *M*, the assumption of the absence of dispersal factors affecting the study region may facilitate the BAM analyses. So, our species model should have accessibility to all the areas of the study region. Secondly, we need to limit the set of factors involved on *A* and *B*. Climatic conditions are a good choice to characterize *A* when we work at large extension and coarse resolution scales (Elith and Leathwick 2009). The *B* factors would be limited to the presence of positively interacting species (a host, prey, or mutualist) or its absence if interacting negatively (i.e., a predator or parasite). Once we have established the theoretical framework and the geographical scale (large extension and coarse resolution for climatic variables characterizing *A*), the next step is to use ENMs to estimate *A* and *B* areas. *A* can be estimated, in a practical way, predicting the climatically suitable areas for the exotic species in the study region, through ENM analysis and using the global occurrence dataset of the species and global climatic information. The estimation of *B* areas can be carried out in the same way, but predicting the climatically suitable (for a positive interaction) or unsuitable (for a negative interaction) areas for the interacting species. Consequently, we will need global occurrence data for these species. Finally, combining both predictions, representing the *A* and *B* areas in the geographical space of our study region, we will be able to highlight the proportion and distribution of the *G*_*BI*_ and *BI* areas that will allow to diagnose which interaction model follow *A* and *B* in our target species, and to identify areas where climatic conditions and/or biotic interactions with other species may be acting as specific barriers against the expansion into those areas.

### Study system: entocytherid ostracods and their host crayfish

Invasive crayfish species are known to cause important harms to the native biota from the invaded site (McCarthy et al. 2006; Matsuzaki et al. 2009; Olden et al. 2011). A well-known impact in Europe was the “spillover” effect caused by the oomycete *Aphanomyces astaci* (Schikora, 1906), carried by American exotic crayfish and becoming one of the main problems for native European crayfish conservation (Gil-Sánchez and Alba-Tercedor 2002). The impact of *A. astaci* on European native crayfish is a typical case of the so-called naive host syndrome: a novel host receiving an exotic symbiont might be severely affected due to lack of history-evolved resistance (Taraschewski 2006; Mastitsky et al. 2010). Crayfishes have a rich associated biota (Edgerton et al. 2002), including entocytherids. The Entocytheridae is an ostracod family constituted entirely by epicommensal species on other crustaceans (Hart and Hart 1974). Entocytherinae, the main subfamily of the group with 183 species, are native from North and Central America living on Cambaridae and Astacidae crayfishes. Recently, two American exotic entocytherid species associated with invasive crayfish were cited in Europe and Japan: *Ankylocythere sinuosa* (Rioja, 1942), found in some localities of the E Iberian Peninsula, associated with *Procambarus clarkii* (Girard, 1852) (Aguilar-Alberola et al. 2012) and *Uncinocythere occidentalis* (Kozloff and Whitman, 1954), cited in a few German and Japanese localities living on *Pacifastacus leniusculus* (Dana, 1852) (Smith and Kamiya 2001; Grabow and Martens 2009; Grabow et al. 2009). In their native range, both entocytherid species have been found in 47 different host species in the case of *A. sinuosa* and three different species of crayfish in the case of *U. occidentalis* (Mestre and Mesquita-Joanes 2013), suggesting that they are not very host specific as seems to be common in the group (Mestre et al. in press). Although both exotic crayfish species have a much longer history in Europe (more than 35 years), entocytherids had not been previously detected, probably because they are tiny (<0.5 mm in length) and apparently not harmful to their hosts. On the other hand, we found no previous comprehensive study, which has checked the presence of Entocytheridae (native or exotic) in European native crayfish.

Exotic entocytherids and crayfishes are particularly adequate to analyze the interactions between *A* and *B* in the geographical space. The total dependence of the entocytherids on their crayfish hosts allows to easily estimate *B* as the crayfish host species presence. Moreover, due to the long invasion history of exotic crayfish in Europe with multiple introduction events by humans in many European countries (Holdich 2002), we can simplify our BAM models assuming the absence of dispersal barriers for these organisms in Europe. Finally, the low host specificity shown by the exotic entocytherids points to the possibility of restriction by host dependence in the invaded range, because they suffer a reduction in host availability from multiple crayfish host species in the native range to just a few exotic crayfish host species in the invaded range.

### Set theory approach: dominance of biotic or abiotic factors in the invasion process?

Symbiont organisms associated with invasive hosts can join them to invaded areas, although a filtering selection in initial invasive stages occurs, as stated by the Enemy Release Hypothesis (Torchin et al. 2003). Having overcome the filters, they must accompany their hosts in the expansive phase. Then two questions arise: Are exotic symbionts able to travel with their hosts wherever they go or could they have physiological limitations preventing them from doing so? Alternatively, could they be limited by their host's tolerances to colonize all the potential areas they are physiologically able to invade (Wharton and Kriticos 2004)? Regarding the last question, the host climatic restrictions are susceptible to constrain the potential distributions of symbiotic organisms in new invaded areas because exotic symbionts often suffer a reduction in host availability from a number of hosts in their native range to just a few or only one invasive host. We can deal with this issue by analyzing the interactions between *A* (as limited to climatic factors) and *B* (reduced to host availability) in the geographical space using the set theory approach. In this context, the three different models of interaction between *A* and *B* proposed above correspond to the different possibilities that we can find in a symbiont–host system. The first model, where *A* includes *B,* would represent a case where the symbiont has broader abiotic tolerance than its host, so its distribution is simply determined by host availability. In contrast, the second and opposite model, where *B* includes *A*, represents a case where the symbiont has a tolerance to abiotic conditions much more restricted than their hosts', facing a climatic barrier to invade a region. Finally, the third and intermediate model outcome with a partial overlap between *A* and *B* represents a case where there is a spatial segregation between both restriction types, affecting different regions of the geographical space.

### Aims and research strategy

To establish an initial evaluation of the distribution of crayfish-living entocytherids in Europe, we carried out the first extensive sampling campaign on native and exotic European crayfish species using specific entocytherid sampling techniques. Furthermore, the main aim of our survey was to evaluate the interactions between abiotic (climatic) and biotic (host availability) factors in geographical space for exotic symbionts, using ENM techniques combined with a theoretical framework based on set theory. To this end, we used as model organisms the exotic entocytherids found in Europe (*A. sinuosa* and *U. occidentalis*) and their hosts (*P. clarkii* and *P. leniusculus*). For each exotic entocytherid species, we carried out the following steps: (1) We established the theoretical framework based on the BAM models proposed by Soberón and Peterson (2005), specifying the model assumptions; (2) we estimated *A* and *B* areas through ENM modeling; (3) we combined the predicted *A* and *B* through a raster operation highlighting the *G*_*BI*_ and *BI* areas, and, finally, (4) we diagnosed the model of interaction between *A* and *B* that followed each entocytherid species analyzed assessing the relative proportion and distribution of *G*_*BI*_ and *BI*.

## Methods

### Field and laboratory methods

In order to evaluate the distribution of crayfish-living entocytherids in Europe, we sampled 12 crayfish species from 93 widely distributed European localities. Eight crayfish species were considered exotic, and four were native to Europe (Table 1). Crayfishes, caught with bait traps or hand nets, were subjected to entocytherid removal protocols based on submerging specimens in anesthetic liquids (carbonated water or chlorobutanol), as discussed and tested in Mestre et al. (2011). In some other cases, we checked the bottom of the container where crayfish were previously preserved in ethanol. Whatever the protocol used, the liquid (carbonated water, chlorobutanol, or ethanol) where crayfishes were submerged was filtered through a 63-*μ*m mesh-sized filter, and the content retained was stored in ethanol. *A posteriori*, these samples were checked in the laboratory under a stereomicroscope, and the entocytherid species found were identified following Hart and Hart (1974). The copulatory apparatuses of selected adult males were drawn using a camera lucida, and SEM and light microscope photographs of adults were also taken to ascertain identifications. Our spatial analyses were mostly focused on both entocytherid species recently found in Europe, *Ankylocythere sinuosa* (Rioja, 1942), cited in association with *Procambarus clarkii* (Girard, 1852) and *Uncinocythere occidentalis* (Kozloff and Whitman, 1954), living on *Pacifastacus leniusculus* (Dana, 1852).

**Table 1:** Summary of crayfish species checked for entocytherid occurrences in Europe. For each species, we indicate its status in Europe (native or exotic), the number of individuals (N crayfish) and localities (N localities) sampled, and the number of sites with presence of entocytherids belonging to species *Ankylocythere sinuosa*, *Uncinocythere occidentalis,* or an unidentified species

Crayfish species	Crayfish status	N crayfish	N localities	*A. sinuosa*	*U. occidentalis*	Unidentified species
*Astacus astacus* (Linnaeus, 1758)	Native	53	6	0	0	0
*Astacus leptodactylus* Eschscholtz, 1823	Native	142	11	0	0	0
*Astacus* sp.	Native	10	1	0	0	0
*Austropotamobius pallipes* (Lereboullet, 1858)	Native	87	5	0	0	0
*Austropotamobius torrentium* (Schrank, 1803)	Native	15	2	0	0	0
*Cherax destructor* Clark, 1936	Exotic	7	1	0	0	0
*Cherax quadricarinatus* Martens, 1868	Exotic	7	2	0	0	1
*Orconectes limosus* (Rafinesque, 1817)	Exotic	103	4	0	0	0
*Orconectes virilis* (Hagen, 1870)	Exotic	48	6	0	0	0
*Pacifastacus leniusculus* (Dana, 1852)	Exotic	183	18	0	9	3
*Procambarus acutus* (Girard, 1852)	Exotic	40	2	0	0	0
*Procambarus clarkii* (Girard, 1852)	Exotic	495	39	28	1	3
*Procambarus fallax* (Hagen, 1870)	Exotic	4	1	0	0	0

### Applying set theory

BAM diagrams were applied by considering *A* the European geographical areas with suitable environmental (climatic) conditions for entocytherid species, *B* the European areas where host presence allows the existence of entocytherid symbionts, and *M* the European accessible areas for the species. It was assumed that: (1) Mobility-related limitations (i.e., physical dispersal barriers) do not exist for entocytherids and crayfishes in Europe. In set theory notation, we can express this assumption as: ((*A* ∪ *B*) ⊂ *M*) ∧ ((*A*_*H*_ ∪ *B*_*H*_) ⊂ *M*_*H*_) (*H* subscripts indicate the parameters related to the host; those without refer to their symbionts). This assumption is based on the long invasion history of both hosts, *P. clarkii* and *P. leniusculus*, in Europe with multiple introduction events by humans in many European countries (Holdich 2002); (2) the only *B* factors considered are the adequate abiotic conditions for host presence, that is, *B* = *A*_*H*_; and (3) the climatic predictors used in the ENM analyses are good estimators of *A* and *A*_*H*_. In this model frame, three possible interactions between *A* and *B* exist (Fig. 1): (1) *A* includes *B*, *B* ⊂ *A,* or (*A*\*B* ≠ ∅) ∧ (*B*\*A* = ∅); (2) *B* includes *A, A* ⊂ *B,* or (*A*\*B* = ∅) ∧ (*B*\*A* ≠ ∅); (3) A partial overlap between both *A* and *B,* (*A*\*B* ≠ ∅) ∧ (*B*\*A* ≠ ∅). Two areas in the models characterize these three cases: *G*_*BI*_ = *A*\*B* are the available geographical areas with favorable environmental conditions, but inappropriate biotic conditions for entocytherids, which in our models were estimated as the climatically suitable areas for the entocytherid but unsuitable for the host, representing those geographical areas where the symbiont is specifically restricted by host availability; *BI* = *B*\*A* areas with unsuitable environmental conditions, but appropriate biotic conditions, estimated in our models as the climatically unsuitable areas for the entocytherid and suitable for the host, representing those areas where the symbiont is specifically restricted by its own climatic tolerances. Consequently, *G*_*BI*_ is present in cases (1) and (3), and *BI* in (2) and (3) (Fig. 1).

### Data sources for the ENMs

#### Occurrence data

The occurrence data for ENM analyses were extracted from three sources: (1) Own data reported in this work; (2) a worldwide database of entocytherid species and their hosts built by Mestre et al. (2012, in press) from published sources; and (3) the Global Biodiversity Information Facility (GBIF; http://data.gbif.org). After checking and cleaning occurrences to remove duplicate and erroneous points, and subsampling oversampled states or countries (i.e., U.K. and Sweden for *P. leniusculus*) following the same protocol as Iguchi et al. (2004), the number of occurrences, representing the global range of the four species studied, was 281 for *A. sinuosa*, 75 for *U. occidentalis*, 266 for *P. clarkii,* and 307 for *P. leniusculus*. We did not use real absences, as suggested by Jiménez-Valverde et al. (2011) because they are conflictive data, among other reasons, due to the difficulty, in most cases, to have a complete certainty that the species is absent, as may occur in entocytherid populations with low prevalences (Aguilar-Alberola et al. 2012).

#### Environmental data

Environmental predictors were restricted to climatic variables, considered more determinant on large extension and coarse resolution scales (Elith and Leathwick 2009). Climatic data were obtained from WorldClim (Hijmans et al. 2005). Datasets at a 5-arcmin resolution were selected. To avoid problems relating to collinearity between predictors (Dormann et al. 2012), only four climatic variables were utilized: minimum temperature of the coldest month (MinT); maximum temperature of the warmest month (MaxT); annual precipitation (AnPrec), and precipitation seasonality (i.e., coefficient of variation, PrecSeas). The selection of the variables was based on the fact that they reflect thermal limits and water environmental availability, consistently relating to important physiological attributes in our organisms, that is, thermoregulation and hydric stress. The effect of these climatic variables on the large-scale distribution of both crayfish species treated is well supported (Capinha et al. 2012). Regarding the entocytherids, both temperature and hydroperiod are important variables affecting the population dynamics of Ostracoda (Mesquita-Joanes et al. 2012), and the strong effects of temperature have been shown in the entocytherid species *A. sinuosa* (Castillo-Escrivà et al. 2013). A previous analysis of collinearity between the selected predictors on our occurrence species data was carried out based on graphical tools from the R (R Core Team 2013) raster package (Hijmans and van Etten 2012) and calculations on correlations between variables. No graphical evidence for collinearity was found, and all the paired combinations of predictors showed an *|r|* < 0.7 in all the climatic datasets for the four species studied.

### ENM analyses

#### ENM modeling

We applied ENMs to predict the climatically suitable areas for each entocytherid species as an estimation of *A* areas of the BAM models, and the climatically suitable areas for each corresponding host species, to estimate *B* areas. The ENMs were built with biomod2 (Thuiller et al. 2013), and the raster management was implemented using raster. Geographical resolution was the same for both models and predictions, determined by the environmental raster data, that is, five arcmin. The extension for the models was global, but European (12°W–60°E; 30°N–75°N) for predictions. ENMs were designed using the world occurrences of each species by also including the invaded range to improve their predictive ability in invaded areas (Broennimann and Guisan 2008; Capinha et al. 2011).

We applied ensemble modeling techniques with worldwide random selection of pseudo-absences (with the same number than the occurrences), data splitting into 70% for model calibration and 30% to test ENMs, and using eight different algorithms: generalized linear model (GLM), generalized additive model (GAM), generalized boosting model (GBM), artificial neural network (ANN), classification tree analysis (CTA), flexible discriminant analysis (FDA), multiple adaptive regression splines (MARS), and random forest (RF). In biomod2, we used the default algorithm parameters. We repeated the modeling process 800 times, combining ten pseudo-absence selections ×8 algorithms ×10 calibrating-testing repetitions obtaining, as a result, 800 individual projections. Afterward, we averaged those individual projections built from the same pseudo-absence selection and calibrating-testing repetition, but different algorithm, obtaining 100 ensemble projections. For this, we applied a weighted average giving more weight to those algorithms with better performance according to the area under the curve (AUC) parameter (Capinha and Anastácio 2011). Finally, the 100 ensemble projections were averaged to get a final consensus projection, showing the probability of species presence in Europe according to the climatic predictors.

#### Assessing ENM performance

Three different aspects of ENM predictive performance were assessed: the performance of the climatic predictors, the test data predictive ability, and the ENM uncertainty. The performance of the predictors was analyzed with generalized linear models (GLMs) of binomial family with a “logit” link function, where the response variable was a dataset with the occurrence data and a pseudo-absence selection, and the explanatory variables were the climatic predictors. The test data fitting assessment was carried out using the AUC parameter, based on receiver operating characteristic (ROC) plots, representing the probability that the classifier (ENM) will rank a randomly chosen positive instance higher than a randomly chosen negative instance (Fawcett 2006), which reflects the relation between true-positive (well-predicted occurrence) and false-positive (absence predicted as presence) prediction rates (Peterson et al. 2011). We tested the effects of the algorithm type on the AUC results, through GLMs with the binomial family and a “logit” link function. ENM predictive uncertainty was also assessed by plotting the SD of the probabilities of species presence of the 100 ensemble projections, as in Capinha and Anastácio (2011).

### Integration of ENM predictions and set theory to estimate the relative importance of abiotic and biotic factors

Once *A* and *B* areas were estimated through the ENM predictions, we combined both areas to obtain the *G*_*BI*_ and *BI* areas for each entocytherid species, used for the diagnosis. For this, the consensus projection for each species was transformed into presence–absence binary data. Threshold selection was based on threshold optimization by the ROC method (Thuiller et al. 2013). Optimized threshold values from the evaluation of the ensemble models were averaged to obtain a consensus threshold per species. Then, we combined the binary consensus projection of each entocytherid (representing *A*) and its respective host (representing *B*) by a subtraction raster operation to highlight the *G*_*BI*_ and *BI* areas for both symbiont species. Finally, we evaluated each case based on the relative proportion and distribution of *G*_*BI*_ and *BI* in the geographical space of Europe.

## Results

### Exotic entocytherids found in Europe

**Figure 2:**
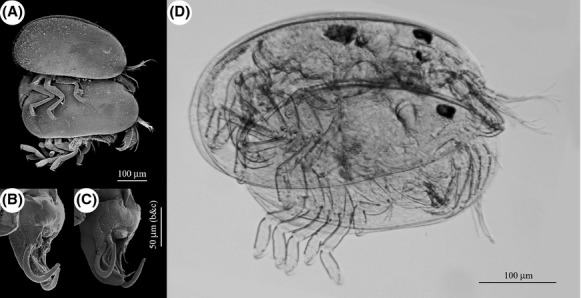
(A–C) Scanning electron microscope (SEM) and (D) stereomicroscope photographs of *Uncinocythere occidentalis* specimens from (A–C) LOC047 and (D) LOC039 (for information about locality codes, see Table S1 in Supporting Information). (A) Mating pair of an adult male (top) and an A-1 female (bottom); (B,C) copulatory organs of adult males in (B) lateral and (C) sublateral views; (D) mating pair of an adult male (top) and an A-2 female (bottom). A-1 refers to the last developmental instar prior to the adult, and A-2 to the juvenile instar prior to A-1.

**Figure 3:**
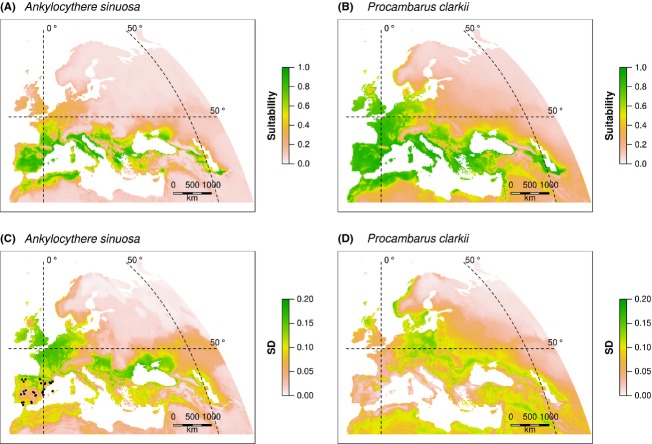
(A,B) Consensus projections obtained from combining the 800 ecological niche models for (A) *Ankylocythere sinuosa* and (B) *Procambarus clarkii* using ensemble modeling techniques, showing the potential climatic suitability for both species in Europe (12°W–60°E; 30°N–75°N). (C,D) Variability among the 100 ensemble projections used to build the consensus projection for (C) *Ankylocythere sinuosa* and (D) *Procambarus clarkii*. Black dots in (C) are localities with *A. sinuosa* occurrences from our field survey. The maps have a 5-arcmin resolution and a Mollweide equal-area projection.

**Figure 4:**
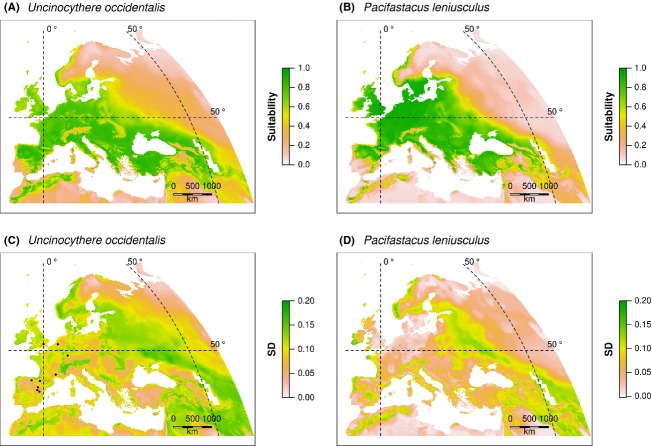
(A,B) Consensus projections obtained from combining the 800 ecological niche models for (A) *Uncinocythere occidentalis* and (B) *Pacifastacus leniusculus* using ensemble modeling techniques, showing the potential climatic suitability for both species in Europe (12°W–60°E; 30°N–75°N). (C,D) Variability among the ensemble projections used to build the consensus projection for (C) *Uncinocythere occidentalis* and (D) *Pacifastacus leniusculus*. Black dots in (C) are localities with *U. occidentalis* occurrences from our field survey. The maps have a 5-arcmin resolution and a Mollweide equal-area projection.

As a result of our field survey (Table 1; see also Table S1 in Supporting Information), two entocytherid species were detected: *Ankylocythere sinuosa* and *Uncinocythere occidentalis*. A general view of a mating pair and morphological details of the latter species is shown in Fig. 2 (Aguilar-Alberola et al. 2012 already presented pictures of exotic populations of *A. sinuosa*). New detailed morphological information on the male copulatory apparatus of some of these European populations of both species is provided in the SI (see Fig. S1). Occurrences of *A. sinuosa* were widely distributed in the Iberian Peninsula (Fig. 3C), while those of *U. occidentalis* were located in NE Iberian Peninsula, Central and N Europe (Fig. 4C). In all cases, host species were *P. clarkii* for *A. sinuosa* and *P. leniusculus* for *U. occidentalis*, with one exception, a locality where *U. occidentalis* was found inhabiting *P. clarkii*, in a small pond in N Spain, where all four species cohabited (LOC039 in Table S1). More specifically, both entocytherid species were found inhabiting the same *P. clarkii* specimen. For the remaining crayfish species sampled, no evidence was found for entocytherid occurrences in any case (Table 1), except for an unidentified entocytherid associated with *Cherax quadricarinatus* Martens, 1868, from a pet shop in Spain (LOC094 in Table S1).

### ENM predictions for the climatic suitability of exotic entocytherids and their hosts in Europe

According to our consensus projections (Figs 3A,B and 4A,B), *A. sinuosa* was the species with the most limited climatically suitable European areas, restricted to circum-Mediterranean regions and some areas around the Black and Caspian Seas (Fig. 3A), while the climatically suitable areas for its host *P. clarkii* included*,* apart from these, a wider region of W Europe (Fig. 3B). In contrast, the climatically suitable areas for *U. occidentalis* and *P. leniusculus* occupied most of Europe, excluding the SW Iberian Peninsula, the highest altitudes of mountain ranges, N Fennoscandia, and the coastal lowlands around the Mediterranean region (Fig. 4A,B).

### ENM assessment

#### Performance of the climatic predictors

According to our GLM results in regard to the effect of the climatic predictors on the probability of species presence (see Table S2), *A. sinuosa* is limited by the lower minimum temperatures (MinT: *Coef*. = 0.006; df = 557; *Z* = 2.422; *P* < 0.05), prefers climates with low annual precipitation (AnPrec: *Coef*. = −0.001; df = 557; *Z* = −2.038 *P* < 0.05), and is negatively affected by precipitation seasonality (PrecSeas: *Coef*. = −0.093; df = 557; *Z* = −10.299 *P* < 0.001), whereas its host, *P. clarkii*, tolerates the extreme temperatures (MaxT: *Coef*. = 0.05; df = 527; *Z* = 2.106 *P* < 0.05, MinT: *Coef*. = −0.012; df = 527; *Z* = −8.067 *P* < 0.001), as well as the precipitation seasonality (PrecSeas: *Coef*. = 0.036; df = 527; *Z* = 8.436 *P* < 0.001). The species *U. occidentalis* shows a preference for lower minimum temperatures (MinT: *Coef*. = −0.005; df = 145; *Z* = −2.171 *P* < 0.05), while its host, *P. leniusculus*, shows limitations in extreme temperatures (MinT: *Coef*. = 0.010; df = 609; *Z* = 7.582 *P* < 0.001, MaxT: *Coef*. = −0.011; df = 609; *Z* = −4.839 *P* < 0.001) and with high precipitation seasonality (PrecSeas: *Coef*. = −0.033; df = 609; *Z* = −7.926 *P* < 0.001). The only GLM with a nonadequate fit according to the residual deviance (*Dev*.) was the model for *U. occidentalis* (*Dev*. = 193.13; df = 145; *P*(>*χ*²)<0.001).

#### AUC and uncertainty assessments

The mean AUC values for those individual ENMs built using the same algorithm were higher than 0.8 in all cases (model types and species), with the highest scores obtained with GAMs (Table 2). The mean AUC values of the ensemble models were higher than 0.95 for all species (Table 2). The GLM results showed that the algorithm type significantly affected the AUC parameter in the ENMs of four species: *A. sinuosa* (*Null Dev*. = 195.99; *Dev. =* 101.03; df = 792; *P*(>*χ²*)<0.001), *U. occidentalis* (*Null Dev*. = 1737; *Dev. =* 1117; df = 792; *P*(>*χ²*)<0.001), *P. clarkii* (*Null Dev*. = 539.55; *Dev.* = 265.91; df = 792; *P*(>*χ²*)<0.001), and *P. leniusculus* (*Null Dev*. = 209.9; *Dev.* = 87.669*;* df = 792; *P*(>*χ²*)<0.001) (see Table S3 for further details on the algorithm effects estimates). However, the model of U. occidentalis has the greater proportion of deviance not explained by the algorithm type (*Dev./Null Dev*.×100 = 64%). In concordance, the SD of the AUC values for the ensemble models showed the highest value in the species *U. occidentalis* (SD = 0.008) (Table 2). Therefore, *U. occidentalis* ENMs presented the greatest predictive instability, according to the AUC assessment.

**Table 2:** Mean and SD values of the area under the curve (AUC) of the 100 individual ecological niche models carried out with the same algorithm for *Ankylocythere sinuosa*, *Uncinocythere occidentalis*, *Procambarus clarkii,* and *Pacifastacus leniusculus*

	GLM		GAM		GBM		ANN		CTA		FDA		MARS		RF		EM	
									
Species	Mean	SD	Mean	SD	Mean	SD	Mean	SD	Mean	SD	Mean	SD	Mean	SD	Mean	SD	Mean	SD
*A. sinuosa*	0.979	0.012	**0.989**	0.006	0.975	0.010	0.953	0.030	0.943	0.020	0.978	0.010	0.981	0.010	0.983	0.009	0.993	0.002
*U. occidentalis*	0.911	0.038	**0.974**	0.024	0.917	0.038	0.881	0.081	0.841	0.056	0.910	0.045	0.907	0.047	0.943	0.030	0.980	0.008
*P. clarkii*	0.919	0.022	**0.950**	0.016	0.935	0.018	0.881	0.039	0.883	0.027	0.923	0.022	0.920	0.023	0.948	0.016	0.968	0.004
*P. leniusculus*	0.966	0.015	**0.984**	0.009	0.974	0.009	0.947	0.020	0.931	0.022	0.970	0.013	0.973	0.012	0.982	0.008	0.991	0.002

The modeling algorithms used were generalized linear model (GLM), generalized additive model (GAM), generalized boosting model (GBM), artificial neural network (ANN), classification tree analysis (CTA), flexible discriminant analysis (FDA), multiple adaptive regression splines (MARS), and random forest (RF). The highest mean AUC values are shown in bold. The last two columns are the mean and SD values of AUC for the ensemble models (EM) used to get the consensus projection for each species.

In the uncertainty assessment, the SD values of the probability of species presence of the ensemble projections remained below 0.2 for all species (Figs 3C,D and 4C,D). *Uncinocythere occidentalis* (Fig. 4C) was the species with more extended areas with higher uncertainty.

### Integration of ENM predictions and set theory to estimate the relative importance of abiotic and biotic factors

**Figure 5:**
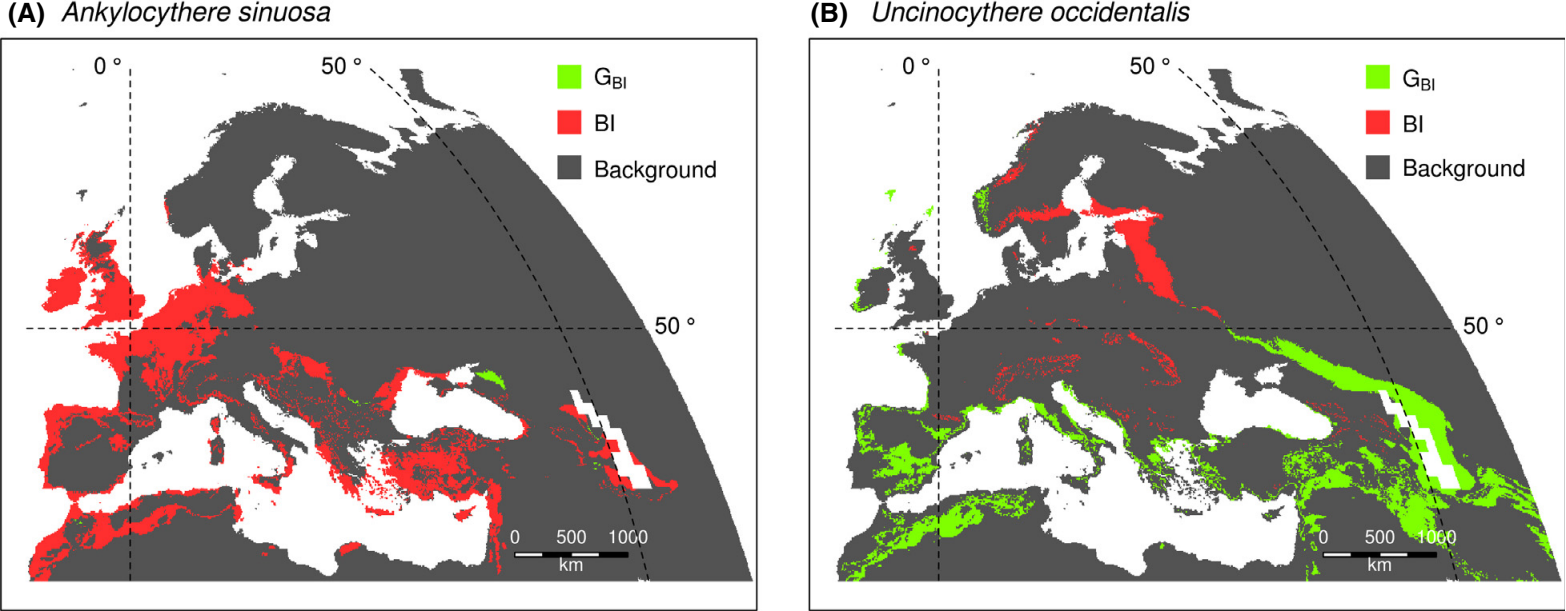
Combined entocytherid-host binary transformed consensus projections for species pairs (A) *Ankylocythere sinuosa* and *Procambarus clarkii,* (B) *Uncinocythere occidentalis* and *Pacifastacus leniusculus*, showing those areas climatically suitable for the symbiont but unsuitable for its host (*G*_*BI*_), and the climatically unsuitable areas for the symbiont and suitable for the host (*BI*), in Europe (12°W–60°E; 30°N–75°N). The maps have a 5-arcmin resolution and a Mollweide equal-area projection (see Fig. 1 and text for definitions of the *G*_*BI*_ and *BI* areas; colors for *G*_*BI*_ and *BI* as in Fig. 1).

Both combinations of entocytherid–host binary consensus projections followed two different patterns (Fig. 5). The *sinuosa*–*clarkii* combination had larger areas with a climatic restriction for the entocytherid (*BI*) in W Europe and circum-Mediterranean regions, with only a few small areas with a restriction by host availability (*G*_*BI*_) around the Black Sea (Fig. 5A). In the *occidentalis–leniusculus* species pair, *G*_*BI*_ occupied a wide range of S European, N African, and Middle East areas, while the *BI* areas appeared mainly in N Europe around the Baltic Sea with some small and diffuse areas in Central Europe associated with the highest altitudes of mountain chains (Fig. 5B). Both symbiont species followed two different distributional patterns of *G*_*BI*_ and *BI* areas, with a predominance of *BI* areas in the case of *A. sinuosa*, closer to the set model where *B* includes *A* (Figs 1B and 5A; case 2 in the Methods section). On the other hand, *U. occidentalis* presented a more balanced proportion of *G*_*BI*_ and *BI* areas, in accordance with a theoretical model with a partial overlap between *A* and *B* (Fig. 1C; case 3 in the Methods section).

## Discussion

In this work, after carrying out the first comprehensive evaluation of the presence and distribution of entocytherids inhabiting crayfishes (exotic and native) in Europe, we were surprised by the low number of species found, which included only two exotic but widely distributed species. For these two species, and according to the main objective of this survey, that is, to compare the influence of biotic and abiotic factors in the spread of invasive symbionts, we analyzed the interactions between their climatically suitable area (*A*) and the suitable area according to host availability (*B*) using ENM techniques in a set theory framework, following Soberón and Peterson (2005). Therefore, for both ostracod symbionts, *A. sinuosa* and *U. occidentalis,* we first estimated their *A* and *B* areas (according to their climate envelopes and their exotic crayfish hosts' *P. clarkii* and *P. leniusculus*) through ENM modeling. The combination of *A* and *B* predictions allowed estimating the *G*_*BI*_ and *BI* areas, which resulted largely different between the two focus species and consequently highlight the importance of both biotic and abiotic factors in the expansion processes of exotic species with tight biological interactions.

### Crayfish-hosted entocytherids in Europe

We evidenced the widely ranging presence of two exotic entocytherid species, *Ankylocythere sinuosa* and *Uncinocythere occidentalis*, in W Europe, previously observed in some locations of E Iberian Peninsula in the case of *A. sinuosa* (Aguilar-Alberola et al. 2012), and in a German locality for *U. occidentalis* (Grabow et al. 2009). Both species have been cited in association with more than one host species in their native range (Mestre and Mesquita-Joanes 2013), including those observed in Europe, *P. clarkii* and *P. leniusculus,* respectively. Notably, both entocytherid species have been observed living on crayfish species belonging to two different families, Cambaridae and Astacidae, showing a broad taxonomic range of hosts. No more entocytherid species were found on the exotic crayfishes sampled in this study across Europe. In contrast, all the sampled American exotic crayfishes had been previously found with entocytherid associates in their native ranges, for example, 27 entocytherid species associated with *Procambarus acutus* (Girard, 1852) (Mestre and Mesquita-Joanes 2013). Moreover, *P. clarkii* and *P. leniusculus* have all been found to be associated with four other entocytherid species (Mestre and Mesquita-Joanes 2013)*.* Our results agree with Torchin et al. (2003) about the effects of strong filters acting on parasites and other symbionts such as entocytherids in early invasive stages.

The absence of native European entocytherids associated with autochthonous crayfish reminds of a similar pattern for another group of crayfish ectosymbionts: the Temnocephalidae. These Platyhelminta are widely distributed in the Neotropical, Ethiopian, Oceanic, and Oriental regions. However, in Europe, a few species are found living as symbionts on cave prawns and shrimps, but not on native crayfish (Gelder 1999). This absence of native ectosymbionts might facilitate the expansion of recently introduced species through host jump given the absence of competitors in their biotic niche. Nevertheless, exotic entocytherids have not been found in native European crayfish hitherto. The main probable reason is that the crayfish plague (*A. astaci*) hinders the coexistence of alien and native crayfish populations because the latter quickly extinguish locally when infected with this parasite. Another additional explanation might rely on the small numbers and high isolation of populations in native crayfish metapopulations, which makes their potential colonization by exotic entocytherids difficult.

### Evaluation of the relative importance of climate and host availability in the geographical space of exotic symbionts

In our models about interactions between climate and host availability in the geographical space of exotic entocytherids in Europe, we showed two species with different distributional patterns of *G*_*BI*_ and *BI* areas. *Ankylocythere sinuosa* had a predominance of *BI* areas, being closer to the model where *A* is included in *B* and therefore this species seems to be mainly restricted by its own climatic tolerances. As shown by the analysis of the predictors, the climatic restrictions of *A. sinuosa* related to *BI* may be due to the limitations to lower minimum temperatures mainly affecting the *BI* areas of W and N Europe, and the precipitation seasonality in *BI* circum-Mediterranean areas. This model suggests the existence of potential invading areas with lower minimum temperatures or higher precipitation seasonality where the host, *P. clarkii*, with a wider tolerance to these climatic variables, could lose its entocytherid symbionts, with the consequent lost of the hypothetical benefit or harm caused by their interaction. On the other hand, *U. occidentalis* has a more balanced proportion of *G*_*BI*_ and *BI* areas, fitting with the model of a partial overlap between *A* and *B*. Both areas affect different European regions, having a spatial segregation of both restriction types at a high scale level. *G*_*BI*_ of *U. occidentalis*, occupying S European, N African, and Middle East areas, may be related to limitations of *P. leniusculus* to higher precipitation seasonality and maximum temperatures. The interpretation of the limitations related to the *BI* areas of *U. occidentalis* is more difficult to ascertain due to the reduced fit shown by the GLM model for the effect of the climatic predictors on the probability of presence of this species. So, in this model of a partial overlap between *A* and *B*, the *G*_*BI*_ areas imply the existence of potential areas that *U. occidentalis* may invade if it is able to jump to other exotic or native crayfish species with a better tolerance to higher precipitation seasonality and maximum temperatures than *P. leniusculus*. This possibility cannot be excluded given the group's low host specificity, that is, one entocytherid species can inhabit more than one host species (Hart and Hart 1974), including crayfish hosts belonging to different families, as was also evidenced in this study in which we found a locality where *U. occidentalis* was also associated with *P. clarkii*, a host having those requirements (tolerance to higher precipitation seasonality and maximum temperatures).

We showed an example of spatial analyses combining ENM and a BAM theoretical framework, applied to the evaluation of the relative importance of climate and host availability in the geographical space of exotic symbionts. Both area types that characterize our models, that is, *G*_*BI*_, where the symbiont is specifically restricted by the host availability, and *BI*, where it is specifically restricted by its own climatic tolerances, apart from their capacity to act as ecological barriers against the symbiont geographical expansion, may have other implications in the invasive process of symbionts and their hosts. In *BI* areas, typical of a model where the symbiont has a tolerance to abiotic conditions much more restricted than their hosts (*B* includes *A*), climatic barriers could act as a host “cleaning” so that the host could lose its symbiont, with the consequent loss of hypothetical benefits or harms derived from such association that may affect the invasive capacity of the host in these areas. On the other side, *G*_*BI*_ areas, characterizing the model where the symbiont has broader abiotic tolerance than its host (*A* includes *B*), may be potentially invaded by the exotic symbiont in the case of a hypothetical host jump to other host species (native or exotic), an event that may derive on a conservation issue threatening the native host species through the “spillover” effects (Roy and Handley 2012; Strauss et al. 2012). Practically, all species have symbiotic organisms affecting them. So, this type of research approach contributes to better understanding the invasive processes and could be applied to conservation plans of native species as potential hosts of exotic symbionts.

In particular, the crayfish–symbiont system has special interest in crayfish conservation. Taking into account the hypothetical jump of exotic entocytherids to European native crayfish, although the main hypothesis for the entocytherid–crayfish relationship is commensalism, this has not been rigorously dealt with, and the line between commensalism and parasitism is often very narrow (Poulin 2007). Moreover, even if it is demonstrated that they are strictly commensal, the role of entocytherids as vectors for parasites and diseases is another possibility that should be considered. Indeed, a rich fauna has been observed in association with ostracods (Mesquita-Joanes et al. 2012), which can act as intermediate hosts of parasites (e.g., Grytner-Ziecina 1996; Moravec 2004). In this sense, we wish to draw attention to the chance of a hypothetical host jump of exotic entocytherids to European native crayfish. Given the low host specificity of entocytherids (Hart and Hart 1974) and the experimentally tested horizontal transfer between adult crayfishes (Young 1971), this jump is quite likely. The potential negative effects of this event on crayfish conservation remain unknown. In this sense, we showed the role of climate and host availability as limiting factors to the expansion of the exotic entocytherid species and identified the new potential areas that the entocytherid could invade if a host jump to native crayfish would occur, information that can be used to get a better assessment of the process.

### Approach limitations and recommendations

An important issue of these methods and, in general, in ENM approaches applied to invasion biology, comes from *A* being calculated by ENMs based on environmental predictors without considering biotic interactions, which are actually modulating the species distribution where those predictors are obtained from. Therefore, we do not estimate *A,* but we actually estimate *A*∩*B*_*GR*_, where *B*_*GR*_ represents the suitable geographical areas for species existence according to all the biotic interactions within the global range (the same applies to *A*_*H*_). For example, our estimation of *BI* for *A. sinuosa* and *U. occidentalis* could be an overestimation of the real *BI* due to the existence of geographical restrictions within their native range caused by competition with other entocytherids, considering that five different species have been found associated with each of both native *P. clarkii* and *P. leniusculus* populations. So in Europe, the lack of competitors would allow the exotic entocytherids to invade part of those overestimated *BI* areas from data obtained mainly from native regions affected by intraspecific competition. In that case, the estimated *A* in our models would actually correspond to the climatically suitable European areas for the entocytherid by considering all the hosts it inhabits and restrictions from competitive interactions with other entocytherids within the global range (*A*∩*B*_*GR*_) (the same may occur in *A*_*H*_). Actually, this is a general issue of ENMs, and in most datasets, environmental effects are confounded with those of competitors and mutualists (Elith and Leathwick 2009). The inclusion of occurrence data from invasive ranges, as we did here, and the design of laboratory experiments about species tolerances against environmental predictors may help to rigorously estimate the *A* areas of the BAM geographical space in order to minimize this problem.

The ENM uncertainty assessment reveals that the *G*_*BI*_ and *BI* geographical areas coincide in most cases with those areas with higher predictive uncertainty (compare Fig. 5 with Figs 3C,D and 4C,D). Probably, the reason is because these areas are usually close to the boundaries of the predicted species distributions, more susceptible to be predictively unstable. Therefore, the estimation of *G*_*BI*_ and *BI* is especially sensitive to ENM accuracy. Consequently, these methods should be based on ENMs with good performance. Along these lines, our ENM assessment based on three ENM performance aspects (i.e., predictors performance, AUC, and uncertainty assessments) give us evidences of weak ENM performance for *U. occidentalis* models: This was the only species with an inadequate fit of the climatic predictors and showed the highest predictive instability according to the AUC assessment through the GLMs (larger proportion of deviance not explained by the algorithm type) and higher ENM predictive uncertainty based on variability shown by the ensemble projections (wider areas with higher variability). These results strongly suggest that our estimation of *G*_*BI*_ and *BI* for this species could be affected by the bad performance of the ENMs for *U. occidentalis*, probably due to the lower number of occurrences available for this species.

As we have shown, a good ENM assessment is essential to analyze the interactions between abiotic and biotic factors in the geographical space. Assessing the performance of the ENM predictors provides useful information about the effects of each individual predictor for each species and can be combined with the results of niche models to better understand which specific variable could be involved on the restrictions present in the different *G*_*BI*_ and *BI* areas. The use of two different approaches to assess ENM performance based on ensemble modeling techniques (i.e., AUC and uncertainty assessments) gives stronger support to our results and, finally, the uncertainty is specially valuable because it helps us to locate those areas with higher predictive instability, and then, we can compare them with the *G*_*BI*_ and *BI* areas to assess the reliability of our estimations.

The methodological approach presented in this work, focused on a symbiont–host system, can also be applied to other systems where the target species is strongly affected by interactions with other species. The range of possibilities may include different kinds of mutualisms, predators with a strong dependence on a specific prey, or species having incompatibilities with the presence of some specific predators, parasites, or competitors. The data required to develop this kind of models are a global occurrence dataset for the interacting species and a global climatic dataset of a large extension and coarse resolution scale. The first step of the analyses through the implementation of set theory is especially important, because it allows a wide variety of theoretical contexts to adapt our models to a particular biological question proposed, for example, the inclusion of dispersal barriers affecting the species expansion through the us*e* of *M*, or the consideration of more than one interacting species to estimate *B*. The generalization of our approach to species without tight biotic relationships would require a higher development of this methodology because, in those cases, the *B* areas do not depend only on the presence of the interacting species, but other parameters would be implied, such as the species densities or the existence of interactions between the environmental conditions and the effect of the biotic interaction. Finally, when applying this kind of models, we do not have to lose the perspective that we deal with dynamic systems (Larson and Olden 2012; Lu et al. 2013).

## Supporting Information

**Figure S1.** Drawings of male copulatory organs of the entocytherid species found during the field survey.

**Table S1.** Detailed European crayfish data checked for entocytherids in this work.

**Table S2.** GLM results for the performance of the climatic predictors.

**Table S3.** GLM results for the fixed algorithm effects on AUC.
