# Supplementary material for: Invasion biology in non-free-living species: interactions between abiotic (climatic) and biotic (host availability) factors in geographical space in crayfish commensals (Ostracoda, Entocytheridae)
Source: Ecol Evol. 2013 Dec 3;3(16):5237–53. doi: 10.1002/ece3.897 (PMC3892332; doi:10.1002/ece3.897)
Supplement: Supplementary file 1 [file ece30003-5237-SD1.doc]

**SUPPORTING INFORMATION**


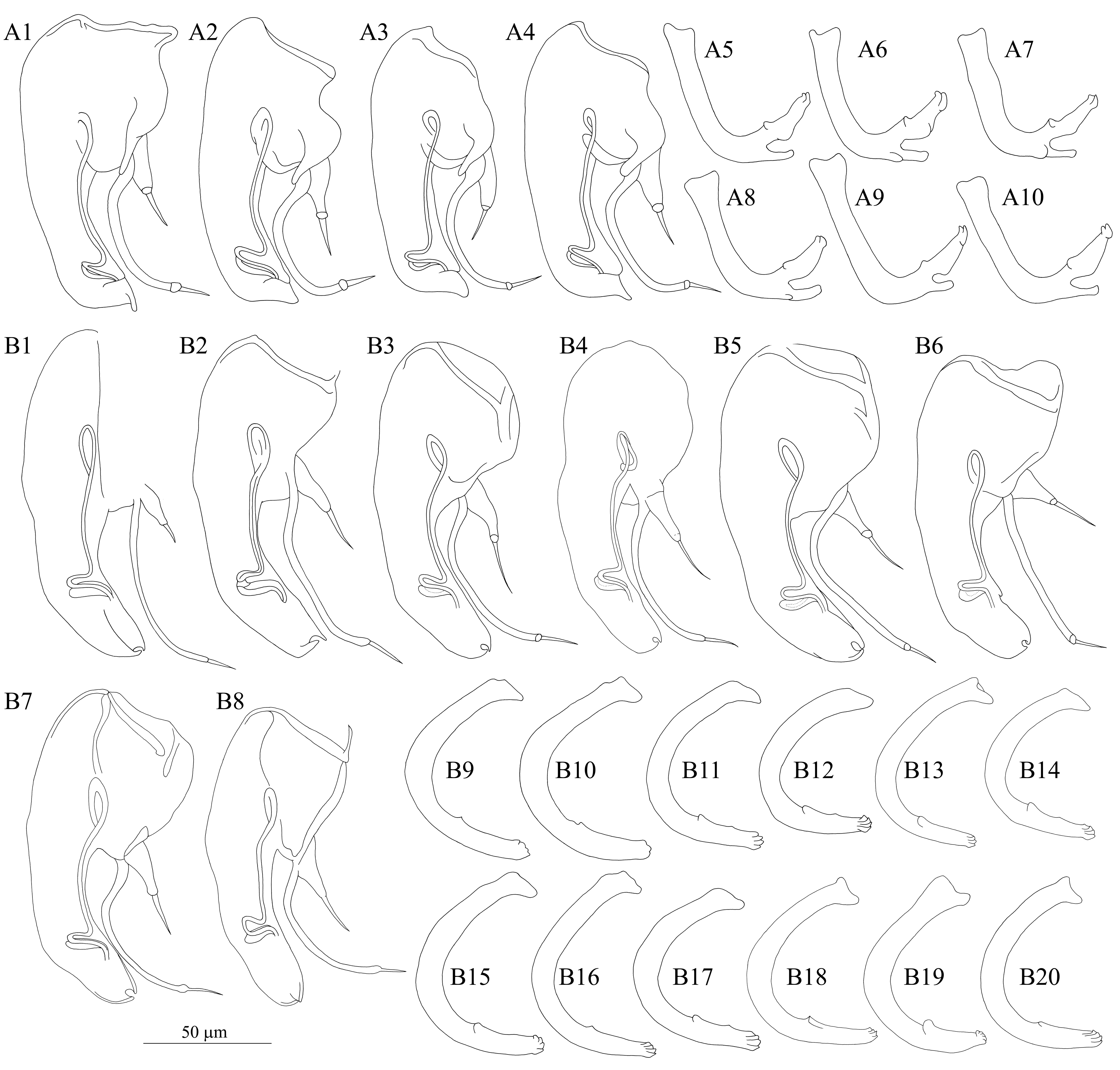
**Figure S1** Variation in male copulatory organs of (A) *Ankylocythere sinuosa* and (B) *Uncinocythere occidentalis* in different European localities from this work, with additional comparative drawings belonging to specimens from the native range and other invasive ranges. (A1-4, B1-8) are drawings of the whole copulatory complex and (A5-10, B9-20) are details of the clasping apparatuses (see Hart & Hart, 1974, for the morphological nomenclature), in lateral views. Specimens A1, A2, A5 and A6 are from Baton Rouge, Louisiana (personal collection); (A3, A8) from LOC027 (Table S1 contains information about locality codes); (A4, A10) from LOC017; (A7) from LOC016; (A9) from LOC005; (B1, B9) are drawings adapted from Hart & Hart (1974) belonging to American specimens; (B2, B10) adapted from Hiruta & Smith (2010) for Japanese exotic specimens; (B3, B11, B12) from LOC041; (B4, B13, B14) from LOC039; (B5, B15) from LOC040; (B6, B16, B17) from LOC044; (B7, B18) from LOC045; (B8, B20) from LOC047; (B19) from LOC046. Note: male copulatory organs are taxonomically relevant as they are used to identify entocytherid species (Hart & Hart, 1974).

**Additional references**

Hiruta, S. & Smith, R.J. (2001) Living freshwater and shallow marine ostracoda in Eastern Hokkaido, Japan (field excursion E). *Guidebook of Excursions of 596 the 14th International Symposium on Ostracoda* (ed. by N. Ikeya), pp. 107-125. The Organising Committee of ISO 2001, Japan.

**Table S1** Detailed data on crayfish samples obtained for checking entocytherid occurrences in Europe carried out in this work. The data fields are: crayfish species; a unique code established to identify each locality; a brief description of the sampling site; name of the sampled locality and state; sampling day; number of individuals caught; crayfish’ European status (native or exotic); the entocytherid species recorded in the locality with the corresponding crayfish species and the protocol used to remove entocytherids from crayfish. In the protocol column, we indicate the time during which crayfish was immersed in the anaesthetic product used. We utilised carbonated water (CW) or chlorobutanol (CB). Alternatively, crayfishes were fixed in ethanol (see Mestre *et al*., 2011, for more detailed information about the protocols used).

| **Crayfish species** | **Locality code** | **Site description** | **Locality** | **State** | **Sampling date** | **Crayfishes sampled** | **Crayfish status** | **Entocytherid species** | **Protocol** |
| --- | --- | --- | --- | --- | --- | --- | --- | --- | --- |
| *Astacus astacus* | LOC087 | River | Mreznica | Croatia | 25/07/11 | 6 | Native | No entocytherids | 5 min CW |
| *A. astacus* | LOC088 | Stream | Kapela Korenicka | Croatia | 07/08/11 | 7 | Native | No entocytherids | 5 min CW |
| *A. astacus* | LOC089 | Stream | Zaklopaca | Croatia | 08/08/11 | 10 | Native | No entocytherids | 5 min CW |
| *A. astacus* | LOC076 | Flooded quarry | Mačkov | Czech Rep. | 22/06/11 | 10 | Native | No entocytherids | 5 min CW |
| *A. astacus* | LOC090 | Pond | Babylon | Czech Rep. | 23/06/11 | 10 | Native | No entocytherids | 5 min CW |
| *A. astacus* | LOC091 | Pond | Hrabice | Czech Rep. | 05/10/11 | 10 | Native | No entocytherids | 5 min CW |
| *Astacus leptodactylus* | LOC075 | River | Duga resa | Croatia | 22/07/11 | 11 | Native | No entocytherids | 5 min CW |
| *A. leptodactylus* | LOC076 | Flooded quarry | Mačkov | Czech Rep. | 22/06/11 | 5 | Native | No entocytherids | 5 min CW |
| *A. leptodactylus* | LOC077 | Lake | Mayaky | Ukraine | 08/09/11 | 20 | Native | No entocytherids | 5 min CW |
| *A. leptodactylus* | LOC078 | Lake | Stanislav | Ukraine | 07/09/11 | 20 | Native | No entocytherids | 5 min CW |
| *A. leptodactylus* | LOC079 | River | Komyshuvatka | Ukraine | 01/09/11 | 19 | Native | No entocytherids | 5 min CW |
| *A. leptodactylus* | LOC080 | Lake | Mayaky | Ukraine | 08/09/11 | 20 | Native | No entocytherids | 5 min CW |
| *A. leptodactylus* | LOC081 | Reservoir | Kerkrade | Netherlands | 05/11/10 | 7 | Native | No entocytherids | Alcohol |
| *A. leptodactylus* | LOC082 | Lake | Karaburun | Turkey | 06/07/11 | 10 | Native | No entocytherids | 5 min CW |
| *A. leptodactylus* | LOC083 | Lake | Durusu | Turkey | 06/07/11 | 10 | Native | No entocytherids | 5 min CW |
| *A. leptodactylus* | LOC084 | Lake | Çakirli | Turkey | 22/07/11 | 10 | Native | No entocytherids | 5 min CW |
| *A. leptodactylus* | LOC085 | Lake | Boyalica | Turkey | 22/07/11 | 10 | Native | No entocytherids | 5 min CW |
| *Astacus* sp. | LOC086 | River | Generalski Stol | Croatia | 23/07/11 | 10 | Native | No entocytherids | 5 min CW |
| *Austropotamobius pallipes* | LOC070 | Stream | Mora de Rubielos | Spain | 05/03/10 | 9 | Native | No entocytherids | 10 min CB |
| *A. pallipes* | LOC071 | Stream | Suera | Spain | 27/07/11 | 22 | Native | No entocytherids | 7 min CB |
| *A. pallipes* | LOC072 | Pond | El Hontanar | Spain | 03/08/11 | 20 | Native | No entocytherids | 7 min CB |
| *A. pallipes* | LOC073 | Pond | Bellestar | Spain | 09/08/11 | 16 | Native | No entocytherids | 7 min CB |
| *A. pallipes* | LOC074 | Crayfish farm | Rillo de Gallo | Spain | 03/11/11 | 20 | Native | No entocytherids | 15 min CW |
| *Austropotamobius torrentium* | LOC092 | Stream | Prijeboj | Croatia | 08/08/11 | 5 | Native | No entocytherids | 5 min CW |
| *A. torrentium* | LOC093 | Brook | Nova Kubice | Czech Rep. | 23/06/11 | 10 | Native | No entocytherids | 4 min CW |
| *Cherax quadricarinatus* | LOC069 | City pond | Wageningen | Netherlands | 02/10/07 | 1 | Exotic | No entocytherids | Live inspection |
| *C. quadricarinatus* | LOC094 | Pet shop | Valencia | Spain | 11/02/09 | 6 | Exotic | Indeterminate | 15 min CW |
| *Cherax destructor* | LOC068 | Pond | Bagüés | Spain | 01/06/10 | 7 | Exotic | No entocytherids | 15 min CW |
| *Orconectes limosus* | LOC053 | Pond | Tutegny | France | 01/10/10 | 33 | Exotic | No entocytherids | Alcohol |
| *O. limosus* | LOC059 | Dam | Lipno | Czech Rep. | 19/10/11 | 10 | Exotic | No entocytherids | 5 min CW |
| *O. limosus* | LOC060 | Ditch | Gorinxem | Netherlands | 20/05/12 | 20 | Exotic | No entocytherids | Alcohol |
| *O. limosus* | LOC061 | Stream | Wageningen | Netherlands | 07/10/12 | 40 | Exotic | No entocytherids | Alcohol |
| *Orconectes virilis* | LOC062 | River | London | England | 01/09/11 | 6 | Exotic | No entocytherids | 15 min CW |
| *O. virilis* | LOC063 | River | London | England | 08/09/11 | 10 | Exotic | No entocytherids | 15 min CW |
| *O. virilis* | LOC064 | Ditch | Boven-Hardinxveld | Netherlands | 20/05/12 | 2 | Exotic | No entocytherids | Alcohol |
| *O. virilis* | LOC065 | Ditch | Oukoop | Netherlands | 28/09/12 | 10 | Exotic | No entocytherids | Alcohol |
| *O. virilis* | LOC066 | Ditch | Oukoop | Netherlands | 14/09/12 | 10 | Exotic | No entocytherids | Alcohol |
| *O. virilis* | LOC067 | Ditch | Kanis | Netherlands | 28/10/12 | 10 | Exotic | No entocytherids | Alcohol |
| *Pacifastacus leniusculus* | LOC039 | Irrigation pond | Undués de Lerda | Spain | 01/06/10 | 3 | Exotic | *Uncinocythere occidentalis* | 15 min CW |
| *P. leniusculus* | LOC040 | River | Cimballa | Spain | 17/06/10 | 1 | Exotic | *U. occidentalis* | Alcohol |
| *P. leniusculus* | LOC041 | River | Ademuz | Spain | 03/08/11 | 20 | Exotic | *U. occidentalis* | 15 min CW |
| *P. leniusculus* | LOC042 | River | Covanera | Spain | 28/04/09 | 1 | Exotic | *U. occidentalis* | Alcohol |
| *P. leniusculus* | LOC043 | River | Río Cuervo | Spain | 25/09/10 | 15 | Exotic | *U. occidentalis* | 15 min CW |
| *P. leniusculus* | LOC044 | River | London | England | 20/09/11 | 10 | Exotic | *U. occidentalis* | 15 min CW |
| *P. leniusculus* | LOC045 | Stream | Tilburg | Netherlands | 14/11/12 | 35 | Exotic | *U. occidentalis* | Alcohol |
| *P. leniusculus* | LOC046 | River | Sant-Jean-du-Gard | France | 28/08/10 | 3 | Exotic | *U. occidentalis* | Alcohol |
| *P. leniusculus* | LOC047 | Stream | Baden-Baden | Germany | 20/07/05 | 30 | Exotic | *U. occidentalis* | Alcohol |
| *P. leniusculus* | LOC048 | Stream | Ponts | Spain | 03/06/10 | 4 | Exotic | Indeterminate sp. | 15 min CW |
| *P. leniusculus* | LOC049 | River | Sta Cruz de Campezo | Spain | 09/06/10 | 7 | Exotic | Indeterminate sp. | Alcohol |
| *P. leniusculus* | LOC050 | Stream | Los Pintanos | Spain | 01/06/10 | 10 | Exotic | No entocytherids | 15 min CW |
| *P. leniusculus* | LOC034 | River | Cimballa | Spain | 17/06/10 | 1 | Exotic | Indeterminate sp. | Alcohol |
| *P. leniusculus* | LOC051 | River | Oliana | Spain | 26/05/10 | 5 | Exotic | No entocytherids | Alcohol |
| *P. leniusculus* | LOC052 | River | Sta Cruz de Campezo | Spain | 09/06/10 | 5 | Exotic | No entocytherids | Alcohol |
| *P. leniusculus* | LOC053 | Pond | Tutegny | France | 01/09/10 | 19 | Exotic | No entocytherids | Alcohol |
| *P. leniusculus* | LOC054 | Brook | Babačka brook | Czech Rep. | 20/11/11 | 10 | Exotic | No entocytherids | 5 min CW |
| *P. leniusculus* | LOC055 | Stream | Losser | Netherlands | 06/02/07 | 4 | Exotic | No entocytherids | Alcohol |
| *Procambarus acutus* | LOC057 | Ditch | Boven-Hardinxveld | Netherlands | 20/05/12 | 20 | Exotic | No entocytherids | Alcohol |
| *P. acutus* | LOC058 | Ditch | Giessenburg | Netherlands | 20/05/12 | 20 | Exotic | No entocytherids | Alcohol |
| *Procambarus clarkii* | LOC001 | Irrigation channel | Oliva | Spain | 28/04/10 | 15 | Exotic | *Ankylocythere sinuosa* | 15 min CW |
| *P. clarkii* | LOC002 | Irrigation channel | Puçol | Spain | 30/04/10 | 20 | Exotic | *A. sinuosa* | 15 min CW |
| *P. clarkii* | LOC003 | Reservoir | Valparaíso | Spain | 06/05/10 | 9 | Exotic | *A. sinuosa* | 15 min CW |
| *P. clarkii* | LOC004 | Lake | Carucedo | Spain | 07/05/10 | 15 | Exotic | *A. sinuosa* | 15 min CW |
| *P. clarkii* | LOC005 | Lake | Chozas de Arriba | Spain | 07/05/10 | 11 | Exotic | *A. sinuosa* | 15 min CW |
| *P. clarkii* | LOC006 | Irrigation channel | Vivares | Spain | 15/05/10 | 20 | Exotic | *A. sinuosa* | 15 min CW |
| *P. clarkii* | LOC008 | Stream | Rosalejo | Spain | 15/05/10 | 5 | Exotic | *A. sinuosa* | 15 min CW |
| *P. clarkii* | LOC009 | Stream | Sarrià de Ter | Spain | 20/05/10 | 20 | Exotic | *A. sinuosa* | 15 min CW |
| *P. clarkii* | LOC010 | Stream | Navarcles | Spain | 20/05/10 | 10 | Exotic | *A. sinuosa* | 15 min CW |
| *P. clarkii* | LOC011 | River | Granollers | Spain | 21/05/10 | 8 | Exotic | *A. sinuosa* | 15 min CW |
| *P. clarkii* | LOC012 | River | Quero | Spain | 26/05/10 | 20 | Exotic | *A. sinuosa* | 15 min CW |
| *P. clarkii* | LOC013 | River | Socuéllamos | Spain | 27/05/10 | 4 | Exotic | *A. sinuosa* | 15 min CW |
| *P. clarkii* | LOC014 | Reservoir | Mequinenza | Spain | 02/06/10 | 20 | Exotic | *A. sinuosa* | 15 min CW |
| *P. clarkii* | LOC015 | Irrigation pond | Alpicat | Spain | 03/06/10 | 20 | Exotic | *A. sinuosa* | 15 min CW |
| *P. clarkii* | LOC016 | Reservoir | Sòller | Spain | 09/06/10 | 11 | Exotic | *A. sinuosa* | 15 min CW |
| *P. clarkii* | LOC017 | Irrigation channel | Puerto Real | Spain | 16/06/10 | 20 | Exotic | *A. sinuosa* | 15 min CW |
| *P. clarkii* | LOC018 | Irrigation channel | Palacios | Spain | 16/06/10 | 20 | Exotic | *A. sinuosa* | 15 min CW |
| *P. clarkii* | LOC019 | Irrigation channel | Padul | Spain | 18/06/10 | 16 | Exotic | *A. sinuosa* | 15 min CW |
| *P. clarkii* | LOC020 | Reservoir | Cubillas | Spain | 18/06/10 | 20 | Exotic | *A. sinuosa* | 15 min CW |
| *P. clarkii* | LOC021 | Lake | Robledo | Spain | 23/06/10 | 11 | Exotic | *A. sinuosa* | 15 min CW |
| *P. clarkii* | LOC022 | Stream | Alcaraz | Spain | 23/06/10 | 1 | Exotic | *A. sinuosa* | 15 min CW |
| *P. clarkii* | LOC023 | Pond | Valencia | Spain | 20/04/11 | 20 | Exotic | *A. sinuosa* | 15 min CW |
| *P. clarkii* | LOC024 | Irrigation channel | Torreblanca | Spain | 19/07/11 | 13 | Exotic | *A. sinuosa* | 15 min CW |
| *P. clarkii* | LOC025 | Irrigation channel | Torreblanca | Spain | 19/07/11 | 20 | Exotic | *A. sinuosa* | 15 min CW |
| *P. clarkii* | LOC026 | Pond | Monroy | Spain | 24/09/11 | 3 | Exotic | *A. sinuosa* | 15 min CW |
| *P. clarkii* | LOC027 | Exotic pet shop | Vodnany | Czech Rep. | 17/06/11 | 33 | Exotic | *A. sinuosa* | 5 min CW |
| *P. clarkii* | LOC028 | Wetland | Delta Ebre | Spain | 10/09/08 | 12 | Exotic | Indeterminate sp. | 15 min CW |
| *P. clarkii* | LOC029 | Irrigation channel | Alberic | Spain | 28/04/10 | 1 | Exotic | No entocytherids | Alcohol |
| *P. clarkii* | LOC030 | Irrigation channel | Sa Pobla | Spain | 09/06/10 | 4 | Exotic | No entocytherids | 15 min CW |
| *P. clarkii* | LOC031 | Stream | Canyamel | Spain | 09/06/10 | 1 | Exotic | No entocytherids | Alcohol |
| *P. clarkii* | LOC032 | Irrigation channel | Sa Pobla | Spain | 10/06/10 | 6 | Exotic | No entocytherids | 15 min CW |
| *P. clarkii* | LOC033 | Irrigation channel | Balazote | Spain | 23/06/10 | 1 | Exotic | Indeterminate sp. | 15 min CW |
| *P. clarkii* | LOC034 | River | Cimballa | Spain | 17/06/10 | 2 | Exotic | Indeterminate sp. | Alcohol |
| *P. clarkii* | LOC035 | Wetland | Torreblanca | Spain | 20/06/10 | 1 | Exotic | No entocytherids | Alcohol |
| *P. clarkii* | LOC036 | River | Bicorp | Spain | 18/11/07 | 4 | Exotic | No entocytherids | 15 min CW |
| *P. clarkii* | LOC037 | City water | Den Haag | Netherlands | 07/08/12 | 21 | Exotic | No entocytherids | Alcohol |
| *P. clarkii* | LOC038 | Stream | Schijndel | Netherlands | 14/10/12 | 30 | Exotic | No entocytherids | Alcohol |
| *P. clarkii* | LOC039 | Irrigation pond | Undués de Lerda | Spain | 01/06/10 | 10 | Exotic | *A. sinuosa* and *U. occidentalis* | 15 min CW |
| *P. clarkii* | LOC007 | Lake | Valdebótoa | Spain | 15/05/10 | 17 | Exotic | *A. sinuosa* | 15 min CW |
| *Procambarus fallax* | LOC056 | Exotic pet shop | Wageningen | Netherlands | 05/10/07 | 4 | Exotic | No entocytherids | Alcohol |

**Table S2** Results of the generalized linear models analysing the effects of climatic predictors on the probability of species presence for *Ankylocythere sinuosa*, *Uncinocythere occidentalis*, *Procambarus clarkii* and *Pacifastacus leniusculus*. The climatic predictors were the maximum temperature of the warmest month (MaxT), the minimum temperature of the coldest month (MinT), annual precipitation (AnPrec) and precipitation seasonality (coefficient of variation, PrecSeas). SE is the standard error. Significant *P* values (<0.05) are marked with the an asterisk.

| **Model** | **Predictor** | **Estimate** | **SE** | ***Z* Value** | ***P*** |
| --- | --- | --- | --- | --- | --- |
| ***Ankylocythere*** | **Intercept** | -4.437 | 1.358 | -3.266 | <0.005* |
| ***sinuosa*** | **MaxT** | 0.028 | 0.004 | 6.905 | <0.001* |
|  | **MinT** | 0.006 | 0.002 | 2.422 | <0.05* |
|  | **AnPrec** | -0.001 | 0.000 | -2.038 | <0.05* |
|  | **PrecSeas** | -0.093 | 0.009 | -10.299 | <0.001* |
| ***Uncinocythere*** | **Intercept** | -1.314 | 1.301 | -1.010 | 0.312 |
| ***occidentalis*** | **MaxT** | 0.003 | 0.004 | 0.958 | 0.338 |
|  | **MinT** | -0.005 | 0.002 | -2.171 | <0.05* |
|  | **AnPrec** | -0.000 | 0.000 | -0.643 | 0.520 |
|  | **PrecSeas** | 0.005 | 0.007 | 0.747 | 0.455 |
| ***Procambarus*** | **Intercept** | -3.981 | 0.775 | -5.133 | <0.001* |
| ***clarkii*** | **MaxT** | 0.005 | 0.002 | 2.106 | <0.05* |
|  | **MinT** | -0.012 | 0.002 | -8.067 | <0.001* |
|  | **AnPrec** | 0.001 | 0.000 | 3.521 | <0.001* |
|  | **PrecSeas** | 0.036 | 0.004 | 8.436 | <0.001* |
| ***Pacifastacus*** | **Intercept** | 5.149 | 0.642 | 8.025 | <0.001* |
| ***leniusculus*** | **MaxT** | -0.011 | 0.002 | -4.839 | <0.001* |
|  | **MinT** | 0.010 | 0.001 | 7.582 | <0.001* |
|  | **AnPrec** | -0.001 | 0.000 | -2.996 | <0.005* |
|  | **PrecSeas** | -0.033 | 0.004 | -7.926 | <0.001* |

**Table S3** Results of the generalized linear models used to assess the effect of algorithm on the area under the curve (AUC) parameter of the 800 individual ecological niche models for *Ankylocythere sinuosa*, *Uncinocythere occidentalis*, *Procambarus clarkii* and *Pacifastacus leniusculus*. The algorithms used for modelling were: artificial neural network (ANN), generalized linear model (GLM), generalized additive model (GAM), generalized boosting model (GBM), classification tree analysis (CTA), random forest (RF), flexible discriminant analysis (FDA) and multiple adaptive regression splines (MARS). SE is the standard error. Significant *P* (<0.05) are marked with an asterisk.

| **Model** | **Algorithm** | **Estimate** | **SE** | **Z Value** | ***P*** |
| --- | --- | --- | --- | --- | --- |
| ***Ankylocythere*** | **Intercept** | -0.048 | 0.005 | -10.625 | <0.001* |
| ***sinuosa*** | **ANN** | 0.000 | --- | --- | --- |
|  | **GLM** | 0.027 | 0.006 | 4.225 | <0.001* |
|  | **GAM** | 0.037 | 0.006 | 5.808 | <0.001* |
|  | **GBM** | 0.023 | 0.006 | 3.593 | <0.001* |
|  | **CTA** | -0.011 | 0.006 | -1.720 | 0.085 |
|  | **RF** | 0.031 | 0.006 | 4.883 | <0.001* |
|  | **FDA** | 0.026 | 0.006 | 4.037 | <0.001* |
|  | **MARS** | 0.029 | 0.006 | 4.503 | <0.001******* |
| ***Uncinocythere*** | **Intercept** | -0.127 | 0.005 | -27.405 | <0.001* |
| ***occidentalis*** | **ANN** | 0.000 | --- | --- | --- |
|  | **GLM** | 0.033 | 0.007 | 5.116 | <0.001* |
|  | **GAM** | 0.100 | 0.006 | 15.521 | <0.001* |
|  | **GBM** | 0.040 | 0.007 | 6.109 | <0.001* |
|  | **CTA** | -0.046 | 0.007 | -6.994 | <0.001* |
|  | **RF** | 0.068 | 0.006 | 10.436 | <0.001* |
|  | **FDA** | 0.033 | 0.007 | 5.028 | <0.001* |
|  | **MARS** | 0.029 | 0.007 | 4.434 | <0.001******* |
| ***Procambarus*** | **Intercept** | -0.127 | 0.005 | -27.401 | <0.001* |
| ***clarkii*** | **ANN** | 0.000 | --- | --- | --- |
|  | **GLM** | 0.042 | 0.006 | 6.440 | <0.001* |
|  | **GAM** | 0.075 | 0.006 | 11.648 | <0.001* |
|  | **GBM** | 0.059 | 0.006 | 9.102 | <0.001* |
|  | **CTA** | 0.002 | 0.007 | 0.278 | 0.781 |
|  | **RF** | 0.073 | 0.006 | 11.250 | <0.001* |
|  | **FDA** | 0.046 | 0.006 | 7.141 | <0.001* |
|  | **MARS** | 0.044 | 0.006 | 6.730 | <0.001******* |
| ***Pacifastacus*** | **Intercept** | -0.054 | 0.005 | -11.987 | <0.001* |
| ***leniusculus*** | **ANN** | 0.000 | --- | --- | --- |
|  | **GLM** | 0.020 | 0.006 | 3.115 | <0.005* |
|  | **GAM** | 0.038 | 0.006 | 5.935 | <0.001* |
|  | **GBM** | 0.028 | 0.006 | 4.420 | <0.001* |
|  | **CTA** | -0.017 | 0.006 | -2.700 | <0.01* |
|  | **RF** | 0.036 | 0.006 | 5.701 | <0.001* |
|  | **FDA** | 0.024 | 0.006 | 3.781 | <0.001* |
|  | **MARS** | 0.027 | 0.006 | 4.170 | <0.001***** |
